# Remote blood pressure management for postpartum hypertension: a cost-effectiveness analysis

**DOI:** 10.1016/j.ajogmf.2024.101442

**Published:** 2024-07-27

**Authors:** Jenny Y. Mei, Alisse Hauspurg, Kate Corry-Saavedra, Tina A. Nguyen, Aisling Murphy, Emily S. Miller

**Affiliations:** Division of Maternal-Fetal Medicine, Department of Obstetrics and Gynecology, University of California, Los Angeles, Los Angeles, CA (Mei, Corry-Saavedra, Nguyen, and Murphy); Division of Maternal-Fetal Medicine, Department of Obstetrics and Gynecology, University of Pittsburgh Medical Center Magee-Womens Hospital, Pittsburgh, PA (Hauspurg); Division of Maternal-Fetal Medicine, Department of Obstetrics and Gynecology, Warren Alpert Medical School of Brown University and Women & Infants Hospital, Providence, RI (Miller).

**Keywords:** cost-effectiveness, hypertensive disorders of pregnancy, postpartum readmission

## Abstract

**BACKGROUND:**

Recognizing the importance of close follow-up after hypertensive disorders of pregnancy, many centers have initiated programs to support postpartum remote blood pressure management.

**OBJECTIVE:**

This study aimed to evaluate the cost-effectiveness of remote blood pressure management to determine the scalability of these programmatic interventions.

**STUDY DESIGN:**

This was a cost-effectiveness analysis of using remote blood pressure management vs usual care to manage postpartum hypertension. The modeled remote blood pressure management included provision of a home blood pressure monitor, guidance on warning symptoms, instructions on blood pressure self-monitoring twice daily, and clinical staff to manage population-level blood pressures as appropriate. Usual care was defined as guidance on warning symptoms and recommendations for 1 outpatient visit for blood pressure monitoring within a week after discharge. This study designed a Markov model that ran over fourteen 1-day cycles to reflect the initial 2 weeks after delivery when most emergency department visits and readmissions occur and remote blood pressure management is clinically anticipated to be most impactful. Parameter values for the base-case scenario were derived from both internal data and literature review. Quality-adjusted life-years were calculated over the first year after delivery and reflected the short-term morbidities associated with hypertensive disorders of pregnancy that, for most birthing people, resolve by 2 weeks after delivery. Sensitivity analyses were performed to assess the strength and validity of the model. The primary outcome was the incremental cost-effectiveness ratio, which was defined as the cost needed to gain 1 quality-adjusted life-year. The secondary outcome was incremental cost per readmission averted. Analyses were performed from a societal perspective.

**RESULTS:**

In the base-case scenario, remote blood pressure management was the dominant strategy (ie, cost less, higher quality-adjusted life-years). In univariate sensitivity analyses, the most cost-effective strategy shifted to usual care when the cost of readmission fell below $2987.92 and the rate of reported severe range blood pressure with a response in remote blood pressure management was <1%. Assuming a willingness to pay of $100,000 per quality-adjusted life-year, using remote blood pressure management was cost-effective in 99.28% of simulations in a Monte Carlo analysis. Using readmissions averted as a secondary effectiveness outcome, the incremental cost per readmission averted was $145.00.

**CONCLUSION:**

Remote blood pressure management for postpartum hypertension is cost saving and has better outcomes than usual care. Our data can be used to inform future dissemination of and support funding for remote blood pressure management programs.

## Introduction

Hypertensive disorders of pregnancy (HDPs) complicate up to 10% of pregnancies in the United States and are associated with significant pregnancy and postpartum complications, including stroke and seizure.^1–3^ Postpartum hypertension (HTN) remains a leading cause of emergency department (ED) visits and readmissions in the United States.^4–6^ Postpartum readmissions have become a focus of quality improvement as readmissions for HTN are considered a marker for poor outpatient management.^5,7,8^ The first week after delivery is the time with the highest risk of postpartum readmission as physiological changes cause blood pressures (BPs) to increase 3 to 7 days after delivery.^7–9^ Previous studies have shown that up to 60% of readmissions occur before a scheduled outpatient visit.^10–12^

Many centers have initiated programs to support postpartum remote BP management (RBPM).^13–16^ These programs generally use a remote BP monitoring application in which all patients with HDP are enrolled and ancillary staff monitor for abnormal BPs and report back to a supervising clinician. Multiple studies have shown a reduction in both ED visits and readmissions for HTN compared with usual care.^13,14^ A recent systematic review concluded that RBPM likely improves the ascertainment of BP and may reduce racial disparities in the completion of BP measurement.^17^ Other recent studies have shown reduced postpartum adverse outcomes and improved BP control with remote BP monitoring compared with controls.^18,19^ In light of this evidence, the Society for Maternal-Fetal Medicine released a special statement on a proposed checklist for the discharge of patients with HDPs, with a designated section addressing remote BP monitoring.^20^ The checklist items include access to a BP cuff and necessary technology, literacy, BP instructions, and guidance on target BPs.^20^

The implementation of RBPMs has raised concerns regarding their costs and effect on healthcare resources. This study aimed to evaluate the cost-effectiveness of determining the scalability of these programmatic interventions. We hypothesized that RBPM is cost-effective compared with usual care.

## Materials and methods

**FIGURE 1:**
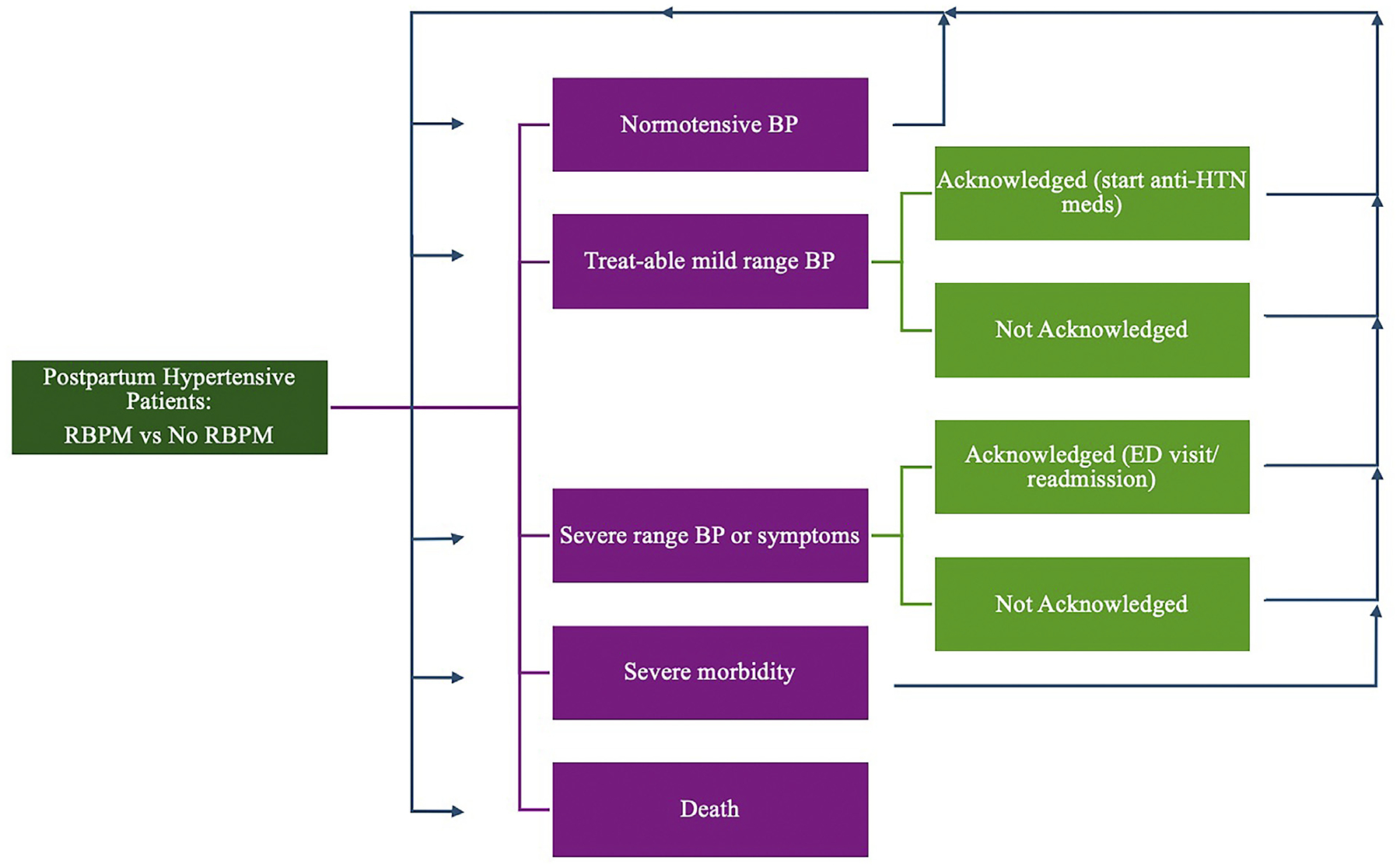
Markov model schematic A schematic of our Markov model that was run over fourteen 1-day cycles to simulate the highest risk time for readmissions and ED visits for postpartum HTN. This is also the period that RBPM was largely used at the primary author’s institution. *BP*, blood pressure; *ED*, emergency department; *HTN*, hypertension; *RBPM*, remote blood pressure management. Mei. Cost-effectiveness analysis of remote postpartum hypertension program. Am J Obstet Gynecol MFM 2024.

We conducted a cost-effectiveness analysis of the use of RBPM vs usual care to manage postpartum HTN. We compared the cost-effectiveness of 2 strategies of monitoring BPs—RBPM and usual care. The modeled RBPM included provision of a home BP cuff, enrollment in a remote BP monitoring program, guidance on warning symptoms, instructions on BP self-monitoring twice daily, and clinical staff to manage population-level BPs during business hours as appropriate. The monitoring program includes software that patients use to upload their BPs for clinician review. Usual care was defined as guidance on warning symptoms and recommendation for 1 outpatient visit for BP monitoring within a week after discharge. We designed a Markov model (Figure 1) that ran over fourteen 1-day cycles to reflect the initial 2 weeks after delivery when most ED visits and readmissions occur and RBPM is clinically anticipated to be most impactful. The perspective of this study was societal. The reporting of cost-effectiveness analysis guidelines was followed throughout the manuscript. Because no human subject was involved in the creation of this model, this study was deemed exempt by the institutional review board of the primary author’s institution.

### Probabilities

The parameter values for the base-case scenario were derived from internal data and literature review. Moreover, published data extracted from the internal electronic medical record (EMR) database were incorporated in certain probability model inputs for the base-case scenario.^13^ Most assumptions, including costs, used existing data from published research or other publicly available studies.

In each strategy (RBPM vs usual care), we assumed the probabilities of each of the following scenarios to occur on any single day: normotensive BPs, mild range BPs, severe range BPs, severe morbidity, and death (Table 1).^2,3,10,13–15,21–41^ We defined mild range BP as a systolic BP (SBP) of 140 to 159 mm Hg or a diastolic BP (DBP) of 90 to 109 mm Hg and severe range BP as an SBP of ≥160 mm Hg or a DBP of ≥110 mm Hg. Severe morbidity was defined as eclampsia, stroke, or intensive care unit admission.

We incorporated the rate of mild or severe range BPs being “acknowledged” in the RBPM arm, meaning the rate at which a logged abnormal BP is reported to the RBPM team and addressed according to predefined algorithms of clinical care. We estimated the probability of an “acknowledged” mild range BP based on the rate of reported adherence with remote monitoring for the RBPM arm vs adherence with standard outpatient monitoring in usual care.^13–15,21,30–32^ We assumed that patients with severe range BPs would have an ED evaluation or readmission if the severe range BPs were recognized. Thus, for severe range BPs, we estimated the probability of an “acknowledged” severe range BP based on the prevalence of symptoms with severe preeclampsia prompting ED evaluation in the usual care arm and added this to the rate of compliance in the RBPM arm.^9,13,33–35^

Different institutions use different protocols to initiate antihypertensive medications; however, we incorporated an estimate of 30% of patients requiring medication adjustment within 14 days after delivery.^13–15,21,30^ We used the Centers for Disease Control and Prevention and World Health Organization reports along with retrospective cohort studies to estimate the rates of death from severe preeclampsia and severe morbidity.^2,3,25,26,33,36,37^ All arms were cycled for 14 days in our Markov model.

### Utilities

The primary clinical outcome of our model was the incremental cost-effectiveness ratio (ICER), which was defined as the cost needed to gain 1 quality-adjusted life-year (QALY). A secondary outcome was the incremental cost per readmission averted. QALYs were calculated over the first year after delivery and reflected the short-term morbidities associated with HDP that, for most birthing people, resolve by 2 weeks after delivery.^9,34^ We used the probability of severe preeclampsia and severe morbidity as markers affecting QALYs for the first 2 weeks after delivery, which is the time with the highest risk of complications. For both usual care and RBPM, we assumed that up to 50% of patients in these cohorts have persistent HTN beyond the initial 6 weeks.^42–46^ Using recent data from the Physician-Optimized Postpartum Hypertension Treatment trial and their substudy that remote monitoring significantly lowers BPs (lower SBP of 6.5 mm Hg and lower DBP of 5.8 mm Hg) and improves cardiac remodeling in the initial year after delivery, we assumed slightly improved QALYs, although not back to baseline in the RBPM arm.^47,48^

### Costs

For RBPM, we included costs associated with using an RBPM program, which included provision of a BP cuff, enrollment in a remote BP monitoring program, and hiring a clinician to oversee the program (modeled as a nurse practitioner).^38–40^ For usual care, we estimated the cost of an outpatient postpartum visit using published data.^41^ Costs were estimated using institutional and online data. RBPM avoids the need for outpatient visits except in cases of readmission; this was incorporated into the costs. For both arms, we included costs of readmission for severe range BPs or severe morbidity. The cost of a nurse practitioner was calculated to include 1.0 full-time equivalent (FTE) for the projected time to oversee an RBPM program. This was projected to cover 900 patients per year based on staffing models of RBPM at the primary author’s institution.

### Analysis

Cost-effectiveness analysis was performed using TreeAge Pro Healthcare (TreeAge Software, Williamstown, MA). Costs and QALYs were calculated for each potential outcome, and the ICER was calculated by dividing the difference in cost by the difference in QALYs. We assumed a willingness to pay (WTP) of $100,000 per QALY to determine cost-effectiveness. Of note, 1-way sensitivity analyses were performed to assess model strength and the thresholds at which the most cost-effective strategy shifted. Moreover, 2-way sensitivity analyses were performed to evaluate the interplay of variables that can vary across different healthcare systems and patient populations: cost of readmission and nursing, probability of abnormal BPs, and rate of “acknowledged” abnormal BPs in RBPM and usual care. Finally, a Monte Carlo simulation was performed to vary all inputs simultaneously by converting all methods into distributions using the base-case and range of each input and then running the analysis over a set number of trials. The variables included in the distributions were the probability of each BP outcome (normotension, mild or severe range BP, severe morbidity, or death), cost of readmissions, costs associated with RBPM (program cost, nursing, and BP cuff) and no RBPM (outpatient visit), and rate of “acknowledged” abnormal BP in both arms. The gamma distribution was used for costs, and the beta distribution was used for all other distributions.

## Results

### Base-case analysis

In the base-case scenario, using RBPM was the dominant strategy (ie, cost less, higher QALYs) (Table 2). The total costs per patient over 14 days were $2987.92 in the RBPM arm and $4213.99 in the usual care arm. There were 15 QALYs and 1% readmission rate over 14 days in the RBPM group vs 14.99 QALYs and 5% readmission rate in the usual care group. The incremental cost per QALY gained in the usual care group would be $630,093.70. Using readmissions averted as a secondary effectiveness outcome, the incremental cost per readmission averted was $145.00.

### One-way and two-way sensitivity analyses

In 1-way sensitivity analyses (Table 3), the most cost-effective strategy shifted to usual care when the cost of readmission was <$2486.89 or when the rate of acknowledgment of severe range BP in RBPM was <0.01. The analysis was not sensitive to the probability of mild or severe range BPs, cost of nursing, or rate of acknowledged mild range BPs.

In 2-way sensitivity analyses, the most cost-effective strategy was sensitive to cost of nurse compared with cost of readmission, cost of readmission compared with rate of acknowledged mild range or severe range BP (both RBPM and usual care), and cost of readmission compared with the probability of normotension, mild range BP, or severe range BP.

### Probabilistic sensitivity analysis

**FIGURE 2:**
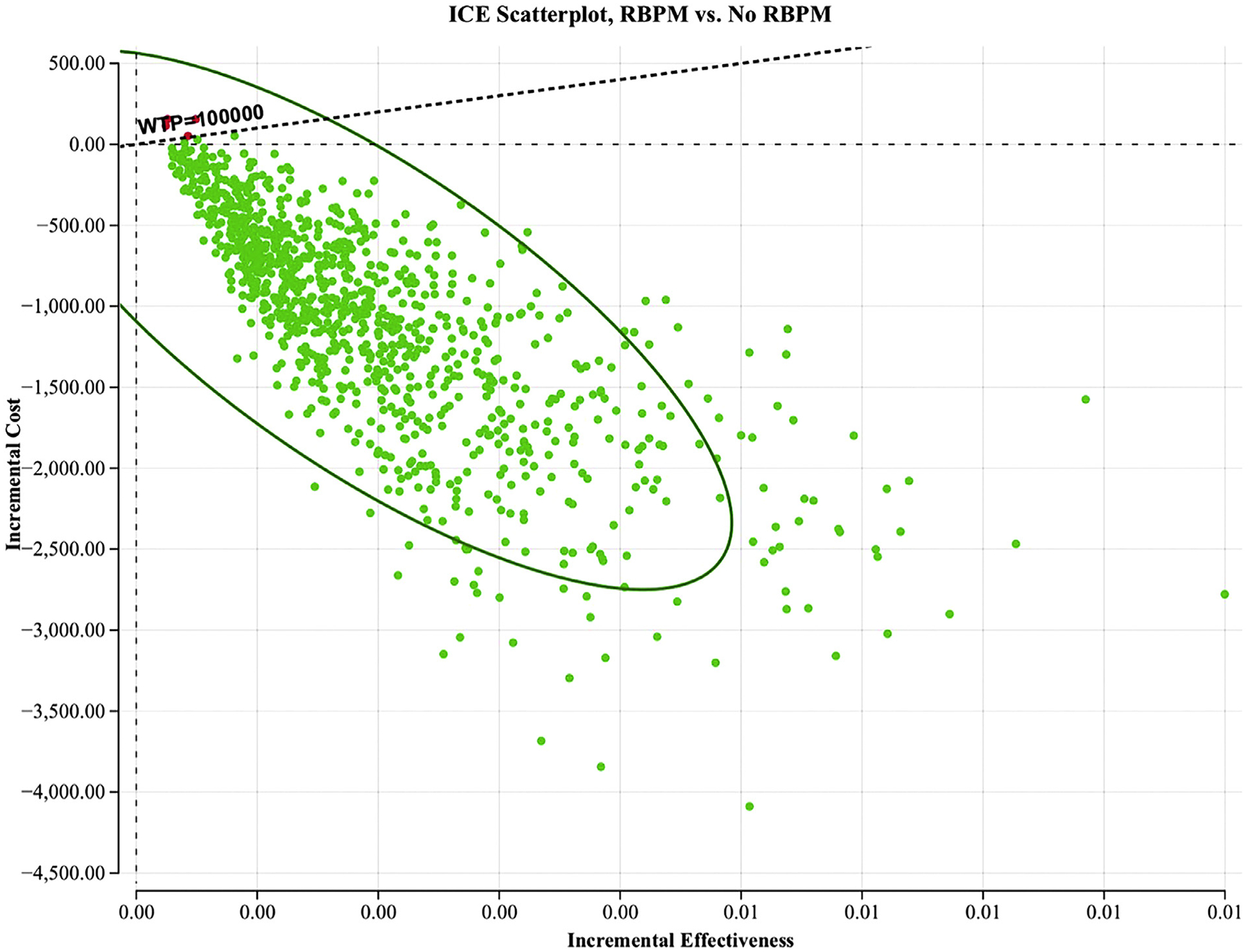
Monte Carlo analysis of 100,000 trials Each *dot* represents a single trial outcome. The *ellipse* represents 95% confidence interval. The *dashed line* represents WTP of $100,000. As shown, 99.28% of points in RBPM lie below this line, which indicates cost-effectiveness. *ICE*, incremental cost-effectiveness; *RBPM*, remote blood pressure management; *WTP*, willingness to pay Mei. Cost-effectiveness analysis of remote postpartum hypertension program. Am J Obstet Gynecol MFM 2024.

In a Monte Carlo analysis of 100,000 patients (Figure 2), assuming a WTP of $100,000 per QALY gained, using RBPM was cost-effective in 99.28% of simulations.

## Discussion

### Principal findings

We found that RBPM for postpartum HTN is cost saving and shows better outcomes than usual care. In 1-way sensitivity analyses, the most cost-effective strategy was sensitive to the cost of readmission and the rate of acknowledged severe range BP in RBPM, although both of these thresholds were well below what would be anticipated in most care settings. RBPM was cost-effective in most simulations in a Monte Carlo analysis.

### Results

A cost-effectiveness analysis performed by Niu et al^49^ used internal data from a parent trial to evaluate the cost-effectiveness of telehealth with remote patient monitoring for postpartum HTN. Compared with “no telehealth,” telehealth was cost-effective and sensitive to the cost of the program, admission cost, and readmission rates.^49^ Our findings corroborate these single-center results while incorporating published data from the literature review.

### Clinical implications

There have been multiple studies demonstrating the importance of remote BP monitoring and management in recent years. Of note, 1 benefit has been a reduction in ED visits and readmissions. A standardized clinical assessment and management plan enacted at 1 institution involving both lowered BP parameters for initiating antihypertensives and outpatient remote monitoring helped decrease ED visits and readmission rates by 80%.^13^ An earlier prospective feasibility cohort study showed no readmission and a 95% retention rate in the program, with an 86% patient satisfaction rate.^50^ In addition, a recent systematic review and meta-analysis corroborated these findings on the reduced rates of postpartum readmission with home BP monitoring compared with conventional office monitoring.^51^ Our analysis demonstrates that, by lowering the rate of readmissions, there is a reduction in cost and morbidity, resulting in RBPM being a dominant strategy despite the associated program costs.

In addition, the effect of RBPM programs was shown in terms of the rate of antihypertensive medication initiation or uptitration after discharge. Multiple studies have shown high rates of medication change, with a range of 20% to 40% of patients requiring medication initiation or uptitration.^13–15,21,31,32,52^ The median number of days for medication initiation was 5 days after delivery, which is only 2 to 3 days after discharge for most patients.^13^ These results support the crucial role of remote BP monitoring programs in improving clinical outcomes and reducing readmissions in high-risk patients. Our results showed that RBPM is the dominant strategy in most clinical scenarios and has an effect in a diverse range of patient scenarios.

Some institutions use lower thresholds for initiating antihypertensive medications, which may affect the rate of severe range BPs diagnosed after discharge.^13,31^ If there is a lower BP threshold to initiate antihypertensives inpatient, there may also be a lower rate of medication initiation or uptitration after discharge through RBPM, as more patients would be discharged on medications. However, some studies have found that patients discharged on antihypertensives were also at higher risk of needing medication uptitration or readmission, which offsets this difference.^7,13^ Regardless, these findings emphasize the need for close outpatient BP monitoring after discharge. Our analysis included studies that used various BP management protocols in both inpatient and outpatient settings, thus making the results applicable across institutions.

We assumed in our analysis that all patients with new-onset severe HTN would have an ED evaluation or readmission given that this is the current clinical practice in most institutions. However, there is an increasing number of institutions, especially those with RBPM programs, that attempt outpatient management of severe range BPs with initiation or uptitration of medications in patients who are asymptomatic. In addition, this shift has been reflected in recent published commentary pieces.^53^ If this were the case, costs would be further reduced, as the readmission rates would decrease, further strengthening RBPM as the dominant strategy.

### Research implications

Although our study focused on the first 2 weeks after delivery, which is the time with the highest risk of maternal morbidity and readmissions, future studies could expand the analysis to 6 weeks after delivery. We did not incorporate the new recommendation for a universal 3-week postpartum visit as this is outside of our 2-week window; however, this recommendation could be included if expanding the analysis to 6 weeks.^20^

Future research could also address the long-term cardiovascular morbidity associated with HDPs. Recent studies have projected a significant effect on cardiovascular health in this population, which is difficult to project.^54^ Previous studies have estimated that approximately 50% of patients with severe preeclampsia subsequently have chronic HTN and up to 8.5% of patients with a history of preeclampsia have a lifetime cardiovascular event.^42–45^ Our model accounted for recent studies showing remote BP monitoring to significantly lower BPs in the first 9 months after delivery^47,48^; however, long-term health effects could show RBPM to be even more cost-effective. For example, the Systolic Blood Pressure Intervention Trial found that intensive SBP control was associated with significantly lower rates of death and cardiovascular disease events than standard SBP control, and this management strategy was shown to be cost-effective.^55–57^

### Strengths and limitations

The strengths of our analysis include the incorporation of a comprehensive literature review alongside institutional RBPM data to best represent how RBPM may function in different healthcare systems. We incorporated adherence to remote monitoring programs into our model, which was roughly consistent across institutions. Although the cost of nursing may vary across different healthcare systems, our univariate analysis showed that the most cost-effective strategy was not sensitive to the cost of nursing. In addition, our univariate analysis was not sensitive to the probabilities of normotension or elevated BPs; thus, the model can likely be applied to most patient populations in different healthcare settings. As remote monitoring gains traction in general, operation costs will likely reduce in subsequent years, which will further improve the cost-effectiveness and augment the results. We incorporated a cost for the RBPM program; however, some institutions have a system linked to the EMR that would be free to use and further lower the RBPM cost.

The limitations of our analysis include variations in readmission costs and how RBPM programs are run across different healthcare systems. Our model was sensitive to the cost of readmission below a very low threshold of $2486.89, which is markedly lower than the cost of most healthcare systems in the United States. Moreover, it was sensitive to the rate of acknowledged severe range BP in RBPM below 1%, which is much lower than what would be expected in RBPM programs. Although our protocol accounted for the cost of a nurse practitioner at 1.0 FTE for the projected time to oversee an RBPM program of 900 patients per year based on staffing models of RBPM at the primary author’s institution, this number should be adjusted based on the patient volume at different institutions. Of note, we have modeled this as the cost per patient of nursing time, which allows for scaling among institutions based on patient volume. Some institutions integrate BP monitoring into a broader program of remote monitoring, such as diabetes mellitus management.^58^ In addition, the costs of the RBPM program and BP cuffs can vary. We assumed the cost of an automated, non–Bluetooth cuff in our base-case analysis. When we reran the model to account for a Bluetooth BP cuff at a presumed cost of $150 per patient,^59^ RBPM remained the dominant strategy in the base-case scenario.

BP management protocols may vary among different studies, as some studies used thresholds for antihypertensive treatment lower than the current American College of Obstetricians and Gynecologists recommendations. These protocols may cause variations in baseline parameter values, although the variation is accounted for in sensitivity analyses. The cost of medications was not included in our model. Given the relatively minor costs of medications (estimated at $90 and $30 for a 1-month supply for nifedipine^60^ and labetalol,^61^ respectively) relative to the readmission costs, we do not anticipate that this change will appreciably change the results of the model. Finally, although our study shows that the RBPM program is a dominant strategy that is not only cost-effective but also beneficial in lowering patient morbidity, it is taken from a societal perspective. For some healthcare systems that generate profit from ED visits and hospital admissions, these outcomes may not be cost-effective from the perspective of the healthcare organization. In its current form, RBPM may be more feasible in a large academic institutional setting, and there may be limitations when applying this model in lower-volume settings.

### Conclusions

We propose that our data be used to inform the future dissemination of RBPM programs. Current financial barriers limit the ability of healthcare systems to fully upscale these programs. Furthermore, the benefits of preventing ED visits and readmissions extend beyond medical costs, and incorporating the costs of childcare and time off from work in these scenarios can improve societal cost savings. In addition, the societal costs of readmissions extend beyond healthcare costs, as there is the emotional cost of infant separation and the potential loss of important time for early infant attachment, with subsequent detrimental potential effects on breastfeeding and mental health.^62^ As healthcare systems provide reimbursements for telehealth, this could further positively influence the cost-effectiveness of RBPM.

Some states have implemented statewide free coverage of remote BP monitoring for chronic HTN.^63^ Furthermore, there is increasing evidence demonstrating improved clinical outcomes with tighter BP control and cost-effectiveness of remote monitoring. In an era of increasing healthcare costs and higher-risk pregnancies, understanding the economic effects of these interventions is of utmost importance.

## Figures and Tables

**TABLE 1:** Parameter values for base-case probabilities and sensitivity analyses

Input (day 1)	Base-case	Range	References
BP range probabilities			
Normotensive BP	0.962	0.95–0.98	13
Mild range BP	0.03	0.02–0.08	13,15,21
Severe range BP	0.008	0.0060–0.0012	22
Severe morbidity (eclampsia or stroke)	0.00007	0.00001–0.00010	23,24
Death	0.000019	0.000019–0.000030	25–28
BP outcome probabilities			
Requiring medication titration by end of 14 d	0.3	0.23–0.62	13–15,21,30
Acknowledged mild range BP in RBPM (daily)	0.65	0.59–0.88	13–15,21,30–32
Acknowledged severe range BP in RBPM (daily)	0.86	0.80–0.95	13,33
Acknowledged mild range BP with no RBPM (daily)	0.04	0.01–0.1	10
Acknowledged severe range BP in no RBPM (daily)	0.60	0.59–0.62	10,34,35
Postpartum readmission for severe HTN with RBPM	0.01	0.00–0.15	13,14,21,32
Postpartum readmission for severe HTN with no RBPM	0.05	0.037–0.150	13–15,30,32
Death from severe preeclampsia	0.00006	0.00004–0.00008	33,36
Death from severe morbidity	0.00006	0.00004–0.00008	2,3,25,26,33,36,37
Cost estimates (USD) per patient			
Readmission	17,549	17,364–17,734	38
Remote BP monitoring program	139	110–150	15
BP cuff	50	40–60	39
Nurse practitioner to oversee program of 900 patients per year at 1.0 FTE, including 30% additional for benefits	$127,374.00/y = $141.53/patient	$101,400.00 – $156,000.00 = $112.67 – $173.33/patient	40
Outpatient BP checkup in usual care	145	100–200	41

Mild range BP = 140 to 159 mm Hg SBP or 90 to 109 mm Hg DBP.

Severe range BP = ≥160 mm Hg SBP or ≥110 mm Hg DBP.

*BP*, blood pressure; *DBP*, diastolic blood pressure; *FTE*, full-time equivalent; *HTN*, hypertension; *RBPM*, remote blood pressure monitoring program; *SBP*, systolic blood pressure; *USD*, United States dollar.

Mei. Cost-effectiveness analysis of remote postpartum hypertension program. Am J Obstet Gynecol MFM 2024.

**TABLE 2:** Cost-effectiveness outcomes based on strategy per patient for base-case

Variable	RBPM	No RBPM
Total cost (USD) over 14 d	$2987.92	$4213.99
Total QALYs over 14 d	15.00	14.99
Readmissions	0.01	0.05
Incremental cost per QALY	—	$630,093.70
Incremental cost per readmission averted	$145.00	—

*QALY*, quality-adjusted life-year; *RBPM*, remote blood pressure monitoring; *USD*, United States dollars.

Mei. Cost-effectiveness analysis of remote postpartum hypertension program. Am J Obstet Gynecol MFM 2024.

**TABLE 3:** Univariate sensitivity analyses evaluating when the most cost-effective strategy shifted to usual care

Baseline parameter	Base estimate	Range	1-way sensitivity threshold
Cost of readmission	$17,549.00	$14,549.00–$20,549.00	<$2486.89
Rate of acknowledged severe range BP in RBPM	0.86	0.72–1.00	<0.01

*BP*, blood pressure; *RBPM*, remote blood pressure monitoring.

Mei. Cost-effectiveness analysis of remote postpartum hypertension program. Am J Obstet Gynecol MFM 2024.

## AJOG MFM at a Glance

### Why was this study conducted?

Given the rapid rise in the use of remote blood pressure monitoring across the country, this study aimed to evaluate the cost-effectiveness of determining the scalability of these programmatic interventions.

### Key findings

Remote blood pressure management for postpartum hypertension is cost saving and has better outcomes than usual care.

### What does this add to what is known?

Our data can be used to inform future dissemination of and support funding for remote blood pressure monitoring programs.
